# Supplementary material for: Embryonic Nicotine Exposure Disrupts Adult Social Behavior and Craniofacial Development in Zebrafish
Source: Toxics. 2022 Oct 15;10(10):612. doi: 10.3390/toxics10100612 (PMC9611253; doi:10.3390/toxics10100612)
Supplement: Supplementary file 1 [file toxics-10-00612-s001.zip › toxics-1920048-supplementary.pdf]

toxics

Supplementary Materials

Embryonic Nicotine Exposure Disrupts Adult Social Behavior and Craniofacial Development in Zebrafish

Gissela Borrego-Soto and Johann K. Eberhart *

Department of Molecular Biosciences, School of Natural Sciences, University of Texas at Austin, Austin, TX 78713, USA
* Correspondence: eberhart@austin.utexas.edu; Tel.: +1-512-232-8340

Figure S1. Behavioral analyses between males and females. No statistical differences were observed between males and females in any of the variables analyzed both in the habituation period and when the live shoal is present. DMSO n = Male 15, Female 7; Nicotine 12.5 µM n = Male 17, Female 11; Nicotine 25 µM n = Male 22, Female 8; Nicotine 50 µM n = Male 13, Female 10.

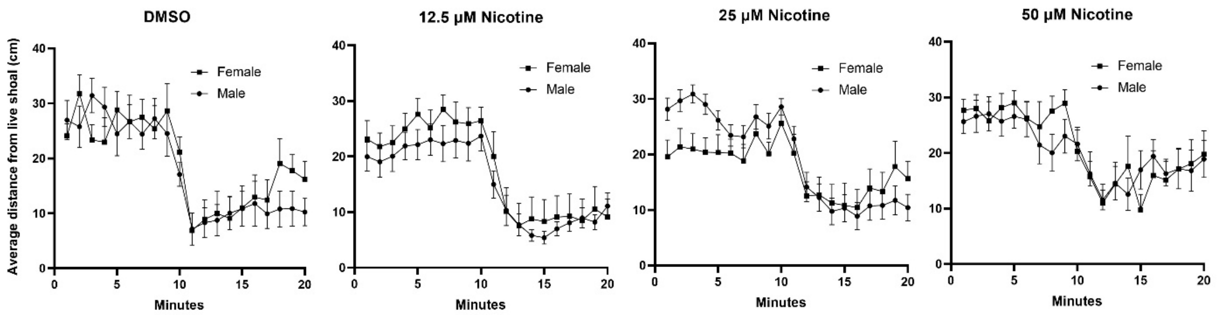

Figure S1. A. Average distance between the experimental fish and the live shoal plotted by minute of the 20-minute behavioral session.

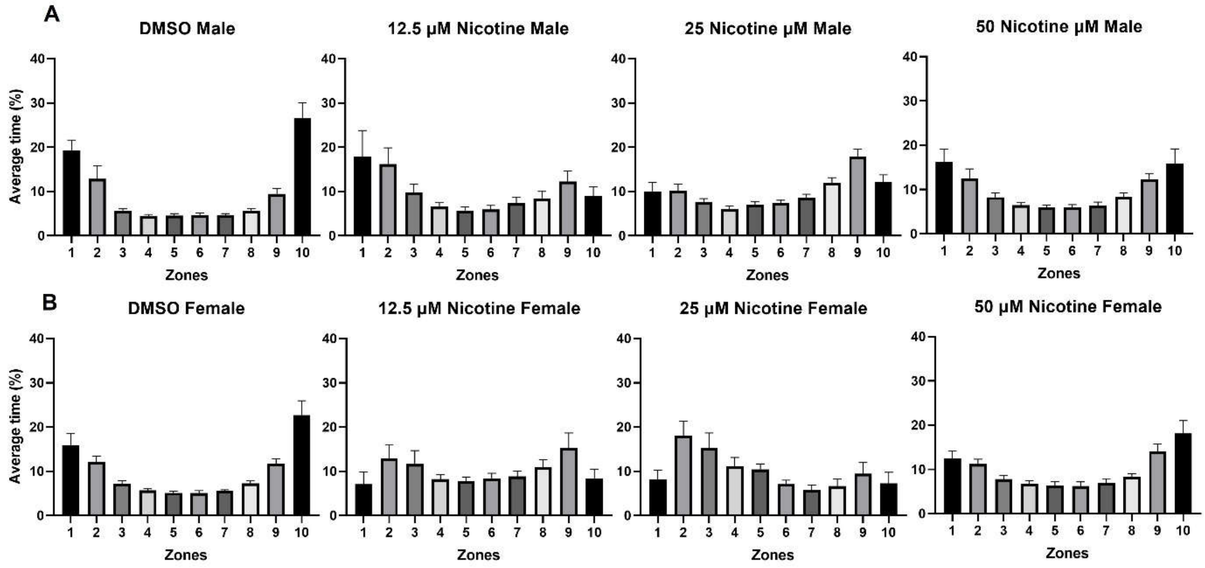

Figure S1. B. Bars represent the time that (A) males and (B) females spent in each of the 10 zones during the first 10 minutes of habituation.

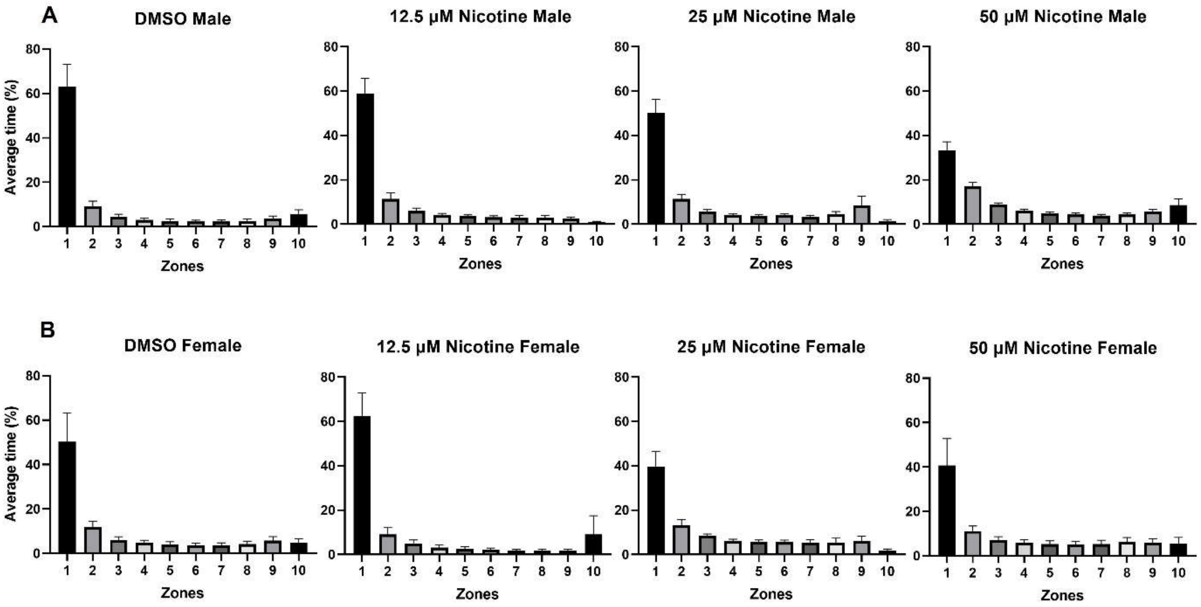

Figure S1. C. Bars represent the time that (A) males and (B) females spent in each of the 10 zones during the last 10 minutes of the behavioral session when the stimulus is visible.

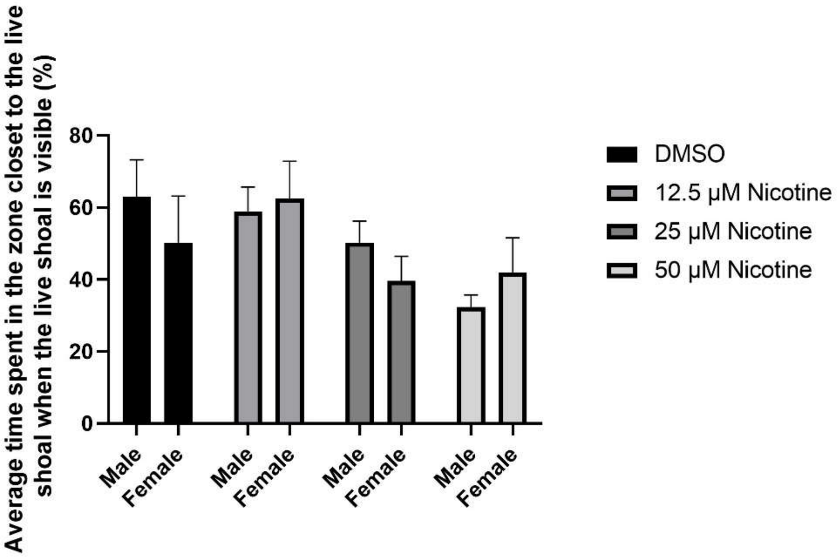

Figure S1. D. Bars represent the average percentage of time spent in the zone closest to the live shoal while the live shoal was visible.

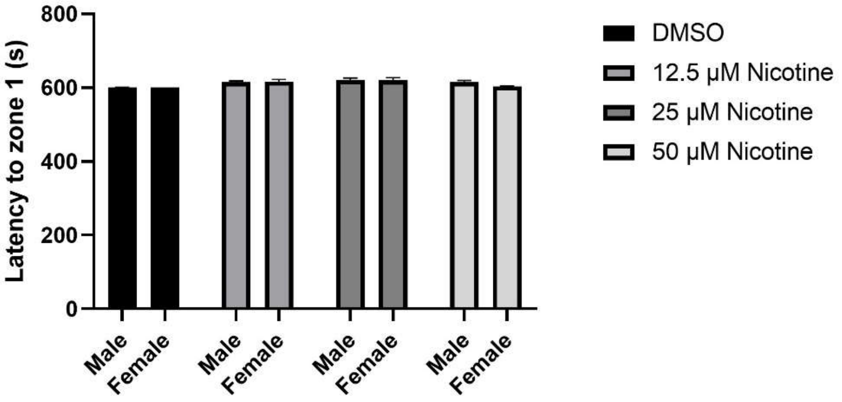

Figure S1. E. Bars represent the time fish take to reach zone 1.

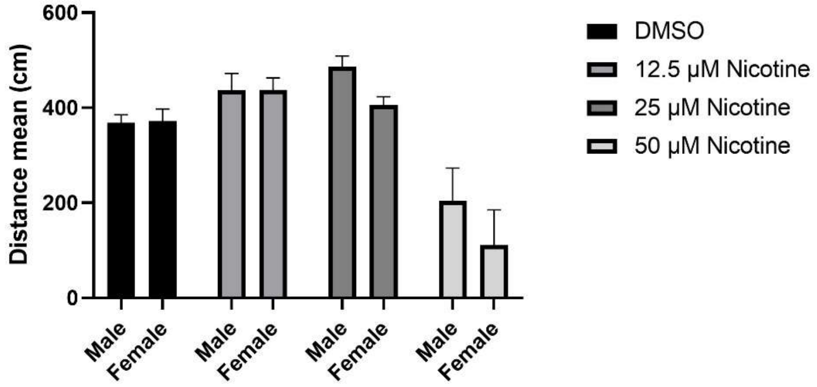

Figure S1. F. Total distance traveled during habituation.

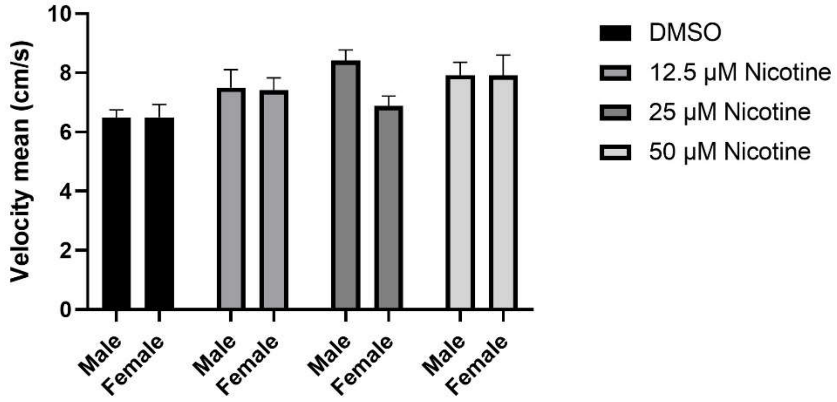

Figure S1. G. Mean velocity during habituation.

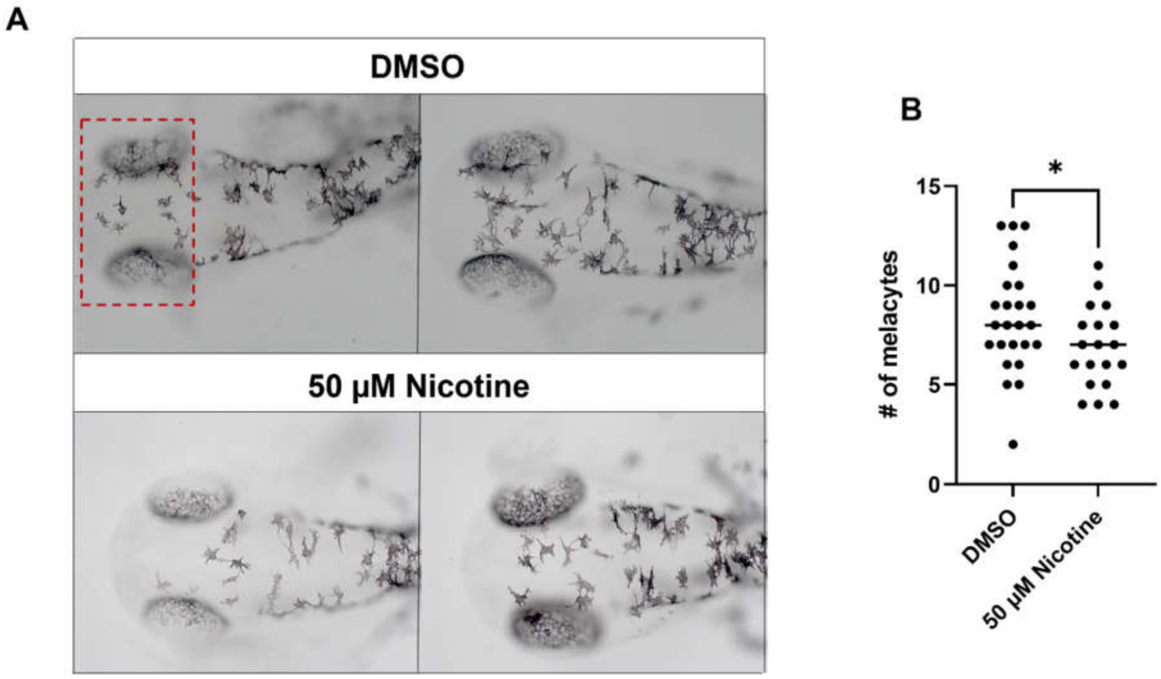

Figure S2. Melanocyte analysis in embryos exposed to DMSO and 50 µM nicotine. Zebrafish embryos were exposed to DMSO and 50 µM Nicotine from 6 to 36 hpf. Yolks were removed and images were collected. (A) Melanocytes were counted manually in the red dotted area. (B) Nicotine exposure caused a statistically significant reduction in the number of melanocytes ($P = 0.0474$; DMSO M = 8.6, SEM ± 0.53; 50 µM Nicotine M = 6.8, SEM ± 0.45). DMSO n = 25; 50 µM Nicotine n = 19. * = $p < 0.05$.

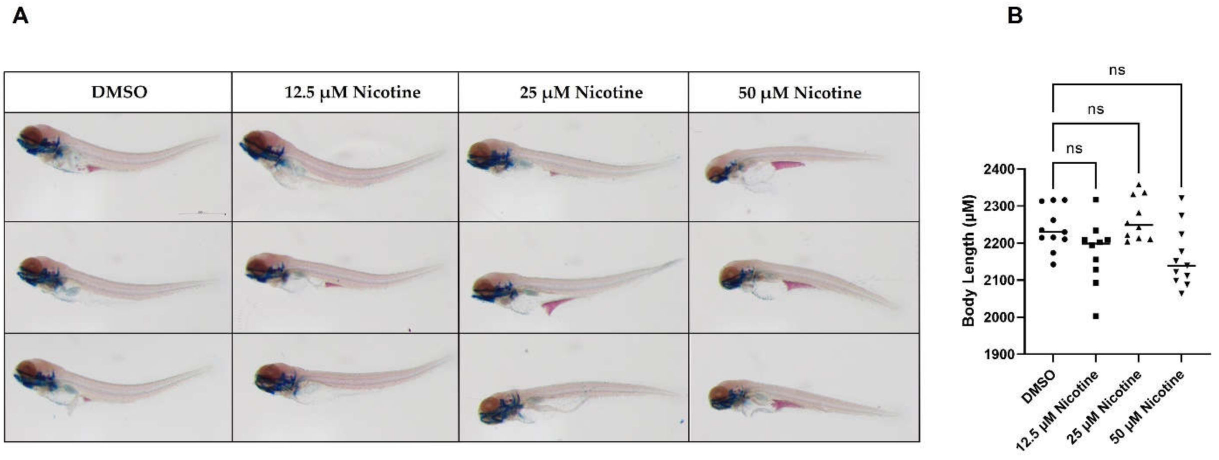

Figure S3. Body Length. (**A**) Total body length was measured at 4 dpf in all experimental groups. Curved bodies were observed at 50 µM Nicotine concentration. (**B**) No significant differences were found in the total body length (DMSO M = 2239.33 µm, SEM ± 16.68; 12.5 µM Nicotine M = 2174.76 µm, SEM ± 25.72, $P = 0.1855$; 25 µM Nicotine M = 2265.66, SEM ± 17.40, $P = 0.8355$; 50 µM Nicotine M = 2161.30, SEM ± 23.32, $P = 0.0684$). DMSO n = 11; 12.5 µM Nicotine n = 10; 25 µM Nicotine n = 10; 50 µM Nicotine n = 11.

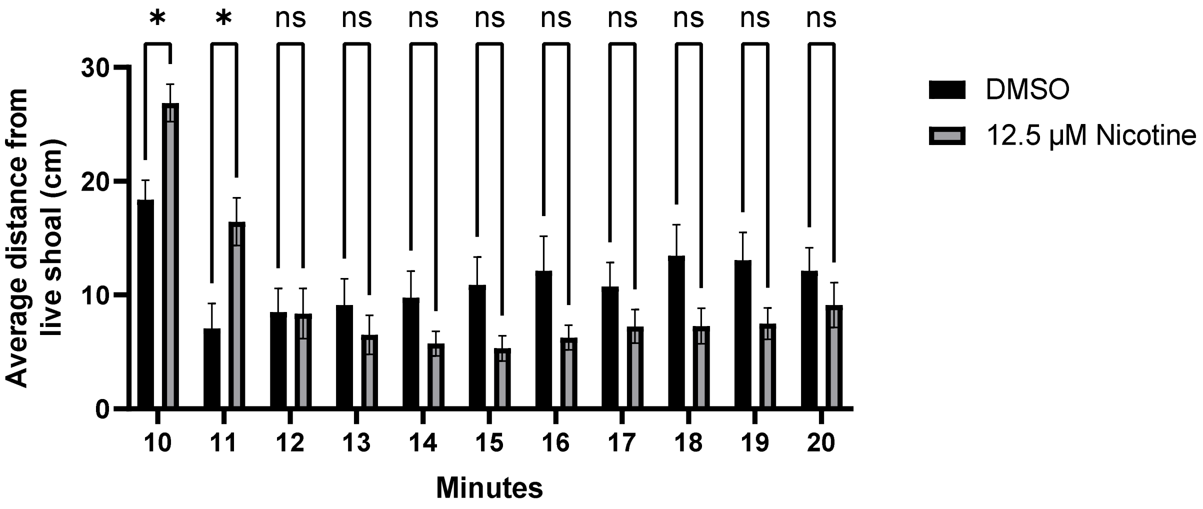

Figure S4. Average distance from the live shoal minute by minute between the DMSO and 12.5 µM Nicotine groups, when the shoal is visible. Statistical differences were only found in the minute 10 and 11 where the DMSO fish is closer to the stimulus than 12.5 µM fish. * = $p < 0.05$.

Table S1. Figure 3. ANOVA P-value summary. * = $p < 0.05$; ** = $p < 0.01$; *** = $p < 0.001$; **** = $p < 0.0001$.

**Meckel's cartilage length**					
Tukey's multiple comparisons test	Mean Diff.	95.00% CI of diff.	Below threshold?	Summary	Adjusted *P* Value
DMSO vs. 12.5 µM Nicotine	2.727	−0.5182 to 5.972	No	ns	0.1321
DMSO vs. 25 µM Nicotine	8.007	4.762 to 11.25	Yes	****	<0.0001
DMSO vs. 50 µM Nicotine	13.47	10.32 to 16.61	Yes	****	<0.0001
12.5 µM Nicotine vs. 25 µM Nicotine	5.28	2.009 to 8.551	Yes	***	0.0003
12.5 µM Nicotine vs. 50 µM Nicotine	10.74	7.567 to 13.91	Yes	****	<0.0001
25 µM Nicotine vs. 50 µM Nicotine	5.461	2.287 to 8.635	Yes	****	<0.0001

**Ceratohyal cartilage length**					
Tukey's multiple comparisons test	Mean Diff.	95.00% CI of diff.	Below threshold?	Summary	Adjusted *P* Value
DMSO vs. Nicotine 12.5 µM	0.9995	−0.6965 to 2.695	No	ns	0.4198
DMSO vs. Nicotine 25 µM	3.961	2.265 to 5.657	Yes	****	<0.0001
DMSO vs. Nicotine 50 µM	7.357	5.713 to 9.002	Yes	****	<0.0001
Nicotine 12.5 µM vs. Nicotine 25 µM	2.962	1.252 to 4.671	Yes	****	<0.0001
Nicotine 12.5 µM vs. Nicotine 50 µM	6.358	4.699 to 8.017	Yes	****	<0.0001
Nicotine 25 µM vs. Nicotine 50 µM	3.396	1.737 to 5.055	Yes	****	<0.0001

**Palatoquadrate cartilage length**					
Tukey's multiple comparisons test	Mean Diff.	95.00% CI of diff.	Below threshold?	Summary	Adjusted *P* Value
DMSO vs. Nicotine 12.5 µM	3.104	0.6457 to 5.563	Yes	**	0.0071
DMSO vs. Nicotine 25 µM	6.671	4.212 to 9.130	Yes	****	<0.0001
DMSO vs. Nicotine 50 µM	9.271	6.887 to 11.65	Yes	****	<0.0001
Nicotine 12.5 µM vs. Nicotine 25 µM	3.567	1.088 to 6.045	Yes	**	0.0015
Nicotine 12.5 µM vs. Nicotine 50 µM	6.166	3.762 to 8.571	Yes	****	<0.0001
Nicotine 25 µM vs. Nicotine 50 µM	2.6	0.1949 to 5.004	Yes	*	0.0286

**Hyosimpletic cartilage lenght**					
Tukey's multiple comparisons test	Mean Diff.	95.00% CI of diff.	Below threshold?	Summary	Adjusted *P* Value
DMSO vs. Nicotine 12.5 µM	1.588	−1.262 to 4.439	No	ns	0.47
DMSO vs. Nicotine 25 µM	10.44	7.591 to 13.29	Yes	****	<0.0001
DMSO vs. Nicotine 50 µM	10.28	7.519 to 13.05	Yes	****	<0.0001
Nicotine 12.5 µM vs. Nicotine 25 µM	8.853	5.979 to 11.73	Yes	****	<0.0001
Nicotine 12.5 µM vs. Nicotine 50 µM	8.695	5.907 to 11.48	Yes	****	<0.0001
Nicotine 25 µM vs. Nicotine 50 µM	−0.158	−2.946 to 2.630	No	ns	0.9988

Table S2. Figure 4. ANOVA P-value summary. * = $p < 0.05$; ** = $p < 0.01$; *** = $p < 0.001$; **** = $p < 0.0001$.

**Neurocranium Length**					
Tukey's multiple comparisons test	Mean Diff.	95.00% CI of diff.	Below threshold?	Summary	Adjusted *P* Value
DMSO vs. 12.5 µM Nicotine	11.21	−3.436 to 25.85	No	ns	0.179
DMSO vs. 25 µM Nicotine	19.45	5.413 to 33.48	Yes	**	0.0042
DMSO vs. 50 µM Nicotine	18.63	5.067 to 32.18	Yes	**	0.0045
12.5 µM Nicotine vs. 25 µM Nicotine	8.238	−6.848 to 23.32	No	ns	0.4513
12.5 µM Nicotine vs. 50 µM Nicotine	7.417	−7.227 to 22.06	No	ns	0.5151
25 µM Nicotine vs. 50 µM Nicotine	−0.8214	−14.85 to 13.21	No	ns	0.9985
**Anterior Neurocranium Length**					
Tukey's multiple comparisons test	Mean Diff.	95.00% CI of diff.	Below threshold?	Summary	Adjusted *P* Value
DMSO vs. 12.5 µM Nicotine	8.635	1.105 to 16.17	Yes	*	0.0203
DMSO vs. 25 µM Nicotine	12.61	5.389 to 19.82	Yes	***	0.0003
DMSO vs. 50 µM Nicotine	11.13	4.154 to 18.10	Yes	***	0.001
12.5 µM Nicotine vs. 25 µM Nicotine	3.97	−3.787 to 11.73	No	ns	0.5064
12.5 µM Nicotine vs. 50 µM Nicotine	2.491	−5.040 to 10.02	No	ns	0.7998
25 µM Nicotine vs. 50 µM Nicotine	−1.48	−8.697 to 5.737	No	ns	0.9418
**Posterior Neurocranium Length**					
Tukey's multiple comparisons test	Mean Diff.	95.00% CI of diff.	Below threshold?	Summary	Adjusted *P* Value
DMSO vs. 12.5 µM Nicotine	3.4	−5.660 to 12.46	No	ns	0.7324
DMSO vs. 25 µM Nicotine	5.771	−2.911 to 14.45	No	ns	0.2841
DMSO vs. 50 µM Nicotine	5.475	−2.913 to 13.86	No	ns	0.299
12.5 µM Nicotine vs. 25 µM Nicotine	2.371	−6.962 to 11.70	No	ns	0.8965
12.5 µM Nicotine vs. 50 µM Nicotine	2.075	−6.985 to 11.13	No	ns	0.9214
25 µM Nicotine vs. 50 µM Nicotine	−0.2964	−8.979 to 8.386	No	ns	0.9997

Trabecula Length					
Tukey's multiple comparisons test	Mean Diff.	95.00% CI of diff.	Below threshold?	Summary	Adjusted *P* Value
DMSO vs. 12.5 μM Nicotine	0.6333	−3.235 to 4.501	No	ns	0.9687
DMSO vs. 25 μM Nicotine	3.693	−0.01416 to 7.399	No	ns	0.0511
DMSO vs. 50 μM Nicotine	0.4825	−3.224 to 4.189	No	ns	0.9838
12.5 μM Nicotine vs. 25 μM Nicotine	3.059	−0.9254 to 7.044	No	ns	0.1761
12.5 μM Nicotine vs. 50 μM Nicotine	−0.1508	−4.135 to 3.834	No	ns	0.9996
25 μM Nicotine vs. 50 μM Nicotine	−3.21	−7.038 to 0.6182	No	ns	0.1231
**Ethmoid Plate Length**					
Tukey's multiple comparisons test	Mean Diff.	95.00% CI of diff.	Below threshold?	Summary	Adjusted *P* Value
DMSO vs. 12.5 μM Nicotine	5.329	−1.395 to 12.05	No	ns	0.1583
DMSO vs. 25 μM Nicotine	10.09	3.592 to 16.58	Yes	**	0.0012
DMSO vs. 50 μM Nicotine	13.29	6.976 to 19.60	Yes	****	<0.0001
12.5 μM Nicotine vs. 25 μM Nicotine	4.759	−1.965 to 11.48	No	ns	0.2379
12.5 μM Nicotine vs. 50 μM Nicotine	7.96	1.413 to 14.51	Yes	*	0.0127
25 μM Nicotine vs. 50 μM Nicotine	3.201	−3.112 to 9.514	No	ns	0.5191
**Ethmoid Plate Width**					
Tukey's multiple comparisons test	Mean Diff.	95.00% CI of diff.	Below threshold?	Summary	Adjusted *P* Value
DMSO vs. 12.5 μM Nicotine	1.742	−1.703 to 5.186	No	ns	0.5125
DMSO vs. 25 μM Nicotine	5.625	2.324 to 8.926	Yes	***	0.0005
DMSO vs. 50 μM Nicotine	5.092	1.647 to 8.536	Yes	**	0.0024
12.5 μM Nicotine vs. 25 μM Nicotine	3.883	0.3346 to 7.432	Yes	*	0.0284
12.5 μM Nicotine vs. 50 μM Nicotine	3.35	−0.3327 to 7.033	No	ns	0.0836
25 μM Nicotine vs. 50 μM Nicotine	−0.5333	−4.082 to 3.015	No	ns	0.9752

Table S3. Average distance from the live shoal minute by minute between the DMSO and 12.5 μM Nicotine groups, when the shoal is visible. Statistical differences were only found in the minute 10 and 11 where the DMSO fish is closer to the stimulus than 12.5 μM fish. * = $p < 0.05$.

Šídák's multiple comparisons test	Mean Diff.	95.00% CI of diff.	Summary	Adjusted *P* Value
DMSO - 12.5 μM Nicotine				
10	−8.500	−16.58 to −0.4170	*	0.0318
11	−9.368	−17.45 to −1.284	*	0.0116
12	0.1338	−7.949 to 8.217	ns	>0.9999
13	2.623	−5.460 to 10.71	ns	0.9922
14	4.023	−4.060 to 12.11	ns	0.8484
15	5.574	−2.510 to 13.66	ns	0.4343
16	5.884	−2.199 to 13.97	ns	0.3543
17	3.492	−4.591 to 11.58	ns	0.9348
18	6.162	−1.921 to 14.24	ns	0.2901

19	5.582	−2.501 to 13.67	ns	0.4319
20	3.023	−5.060 to 11.11	ns	0.9762
